# Acupuncture May Stimulate Anticancer Immunity via Activation of Natural Killer Cells

**DOI:** 10.1093/ecam/nep236

**Published:** 2011-03-10

**Authors:** Michael Francis Johnston, Elizabeth Ortiz Sánchez, Nikola L. Vujanovic, Wenhui Li

**Affiliations:** ^1^Department of Medicine, University of California, USA; ^2^Division of Surgical Oncology, Department of Surgery, University of California, Los Angeles, CA, USA; ^3^University of Pittsburgh Cancer Institute, Departments of Pathology and Immunology, University of Pittsburgh, Pittsburgh, PA, USA; ^4^Department of Chemistry, University of California, Los Angeles, CA, USA

## Abstract

This article presents the hypothesis that acupuncture enhances anticancer immune functions by stimulating natural killer (NK) cells. It provides background information on acupuncture, summarizes the current scientific understanding of the mechanisms through which NK cells act to eliminate cancer cells, and reviews evidence that acupuncture is associated with increases in NK cell quantity and function in both animals and humans. The key contribution of this article involves the use of cellular immunology and molecular biological theory to interpret and synthesize evidence from disparate animal and human studies in formulating the ‘acupuncture immuno-enhancement hypothesis': clinicians may use acupuncture to promote the induction and secretion of NK-cell activating cytokines that engage specific NK cell receptors that endogenously enhance anticancer immune function.

## 1. Introduction

Compelling research findings demonstrate that acupuncture reduces the incidence of chemotherapy-induced acute vomiting [1]. Suggestive findings show that clinicians could use acupuncture to potentially manage cancer-related pain of articular and soft tissue origin [2–7]. Promising evidence suggests that acupuncture relieves fatigue in cancer patients and survivors [5, 6, 8, 9]. Preliminary results are accumulating that acupuncture alleviates cancer-related neurological issues, breathlessness, hot flashes and xerostomia [5–7]. In sum, there are substantial empirical grounds to assume that clinicians could use acupuncture to help patients better tolerate conventional cancer therapies by reducing associated side-effects [1–8]. Further, the evidence is very strong that acupuncture is an extremely safe therapy [10]. In short, the research implies that acupuncture is a helpful, versatile and safe treatment modality for patients with cancer.

Despite potential benefits, however, the conventional oncology community has shown itself reluctant to integrate acupuncture and other complementary and alternative medicine (CAM) modalities into their treatment plans for patients with cancer [11]. One concern is that symptoms treated with CAM modalities are often transitory, which opens the possibility that symptomatic relief may be due to spontaneous remission rather than resulting from a CAM intervention [12–14]. A second concern is that the current procedures of disclosure may undermine informed consent and thereby compromise patient autonomy in such a way that produces negative consequences for conventional therapeutic procedures [15]. A third and related concern is that the scientific community lacks both the scientific evidence and biomedical understanding to reasonably suppose that CAM procedures may increase either survival or disease-free survival of patients with cancer [12–14].

We address these concerns by harnessing theory from cellular immunology and molecular biology to interpretive and synthesize disparate empirical studies into a hypothesis about specific mechanisms through which acupuncture enhances anticancer immune function with implications for prevention and management of the cancer. Specifically, we hypothesize that clinicians may use acupuncture to increase the cytotoxic activity of natural killer (NK) cells by promoting cross-talk between the neurotransmitter network and immune system that is [1] orchestrated by nitric oxide, *β*-endorphins and cytokines and [2] anchored by opioid and NK cell receptors. We refer to this as the “acupuncture immuno-enhancement hypothesis”. For reasons we describe below, we think that one acupuncture point is pivotal although we also expect that treatment protocols involving synergistic points will produce better results. But this detail should not distract the reader from our overall theme: acupuncture can be employed to improve the function and level of NK cells. Our main theme is of great importance to patients with cancer (and the conventional providers who treat them) because NK cells are immune cells known to play a key role in directly killing cancer cells and regulating anticancer immune functions [16–18].

To develop the hypothesis that acupuncture enhances endogenous anticancer immune functions with implications for molecular-level carcinogenic control, we begin with a primer on acupuncture and a review of studies that have investigated the impact of acupuncture stimulation on NK cell function in animals and humans. We then provide an overview of the advancing molecular biological understanding of NK cells. Our key contribution follows in the subsequent section, where we harness cellular immunology and molecular biological theories to interpret and integrate disparate research findings in formulating a hypothesis. Specifically, we hypothesize that acupuncture stimulation enhances NK cell stimulating cytokines and NK cell anticancer cytotoxic activity and proliferation through specific cellular and molecular mechanisms, thus enhancing host resistance to cancer in both rodents and humans. As a finale, we assess the evidential basis of our hypothesis and point to fresh research frontiers.

## 2. Acupuncture: Three Clinically Important Acupoints

Acupuncture encompasses a heterogeneous set of needling methods by which practitioners induce biologic responses that activate pathways in the peripheral and central nervous systems [19]. Conventionally, acupuncture involves insertion of a very thin needle into acupoints and is sometimes accompanied by thrusting and/or twirling. One specific type of acupuncture involves transmitting weak pulses of electrical current through acupuncture needles inserted into acupuncture points. In some instances, the distinction is clinically relevant. For example in the case of chemotherapy-induced acute vomiting [1], the evidence indicates that acupuncture administered with electricity (electroacupuncture) is effective although acupuncture without electricity (manual acupuncture) is not. Acupoints are in the vicinity of peripheral nerves and their bifurcations, neuromuscular attachments, blood vessels and ligaments [20]. The World Health Organization identifies 361 precisely-specified anatomically distinct sites known as acupoints [21].

Three of the most commonly used acupoints are Stomach 36 (ST36, in Chinese *Zusanli*), Pericardium 6 (PC6, in Chinese *Neiguan*) and Large Intestine 4 (LI4, in Chinese *Hegu*). PC6, known as the “antinausea acupoint”, is located between the tendons of palmaris longus and flexor carpii radialis at 2 body-inches (a body-inch or a *cun* is the greatest width of a patient's thumb at the distal phalanx) above the wrist crease [22]. Stimulation of PC6 is found to be beneficially associated with the prevention and relief of nausea and/or vomiting in three separate reviews published by the Cochrane collaboration, a highly prestigious international initiative that involves the application of a rigorous process to systematically review the effects of biomedical interventions in randomized controlled trials across all areas of health care [23, 24]. The reviews are based on evidence from clinical trials, many of which are focused specifically on PC6, that involve thousands of patients who experienced relief of nausea and/or vomiting associated with surgery [25], chemotherapy [1] or pregnancy [26]. Furthermore, a recently published randomized controlled trial found acupuncture as effective as commonly used antiemetic drugs in relieving nausea [27].

LI4 is the most commonly used analgesic acupoint, especially for neck and head pain; it is known as the “antipain acupoint” [28]. LI4 is located at the highest point on the adductor pollicis muscle between the thumb and the index finger. Authors of a 2009 Cochrane review analyze 11 trials with 2317 patients to conclude that the evidence indicates that acupuncture would be a valuable non-pharmacological tool in patients with frequent episodic or chronic tension-type headaches [29]. In a separate 2009 Cochrane review, authors review 22 trials with 4419 patients, to conclude that available studies indicate that acupuncture is at least as effective as, or possibly more effective than, prophylactic drug treatment, and has fewer adverse effects and that therefore acupuncture should be considered as a treatment option [30]. Pragmatic (practice-oriented) research shows acupuncture to be a cost-effective treatment for headaches [31].

In acupuncture clinics, providers most commonly needle ST36 to enhance immune functioning [32]. For this reason, we term ST36 as the “immuno-enhancing acupoint”. ST36 is located 5 cm below the patella and 2 cm lateral of the anterior crest of the tibialis anterior muscle [33, 34]. This review makes the case that one way in which acupuncture enhances immune function is through the modulation of NK cells.

## 3. Suggestive Evidence that Acupuncture Stimulates NK Cells

Limited published studies provide suggestive evidence that acupuncture induces an increase of NK cell activity both in animals and humans. In rats, researchers have shown that carrying out acupuncture on the ST36 acupoint (0.5–1 h a day for 2-3 days) induces, in the spleen, significant increases of NK cell tumoricidal activity and secretion of interleukin-2 (IL-2) and interferon-*γ* (IFN-*γ*) [35]; and parallel increases in expression of the NK cell receptor CD94, protein tyrosine kinase (PTK) and adhesion molecule VCAM-1 genes, and decreases of protein tyrosine phosphatase (PTP) and SHP-1 genes that are critically involved in regulation of NK cell activity [36]. Importantly, researchers have demonstrated that acupuncture stimulation of the ST36 acupoint increases the suppressed NK cell activity in surgically traumatized rats [37].

In humans, Arranz and colleagues in Spain carried out a clinical trial comparing peripheral blood NK cell cytotoxic activity in 36 female patients suffering from high levels of anxiety before and after acupuncture to 20 healthy controls [38]. At baseline, women with anxiety had NK cell activity 3-fold lower than healthy controls (*P* < .0001). A fraction of the patients were then administered one manual acupuncture treatment with needles retained in place for 30 min at ST36 and several other points (SI3, HT3, HT5, LI11, PC6, LI4, TH5, CV3, CV4, CV6, CV15, GB34, GB43, SP6, LV2, UB60, KD6, GV20). Immediately and also at 72 h following the treatment, NK cell activity in the anxious women was still significantly lower than that of the healthy controls (*P* < .01, *P* < .05), but it progressively and significantly increased relative to the patient's baseline levels (*P* < .05, *P* < .0001), respectively. Remarkably, 1 month after finishing a series of 10 acupuncture treatments, the anxious women showed a complete restoration of NK cell cytotoxic activity, which became the same as that in the healthy controls.

Yamaguchi and colleagues in Japan carried out a clinical trial with 17 healthy volunteers from whom they drew blood from a forearm vein 1 h before and 1, 2 and 8 days after treatment [39]. Acupuncture treatment was manual and consisted of insertion of a needle for 5 s into ST36 and a few other points, such as BL18, BL20 and BL23. The scientists found that patients exhibited progressive and statistically significant increases in the percentage of circulating NK cells subsequent to acupuncture treatment at days 1–8 (compared to baseline). Increased NK cell levels were evidenced by counts of both low affinity Fc*γ* receptor (CD16) and neural cell adhesion molecule (NCAM, CD56) positive peripheral blood mononuclear cells (Table 1). In the same study, authors found a respective 7- and 9-fold increases in the number of IFN-*γ* producing peripheral blood cells subsequent to treatment at days 1 and 8 relative to pretreatment. As NK cells are the major immune cells that rapidly produce IFN-*γ* in response to “danger signals” and IFN-*γ* is an important activator of the immune mechanisms that eliminate cancer cells; we specifically reference this finding below in the course of formally developing our hypothesis. 

Petti and colleagues in Italy carried out a clinical trial on 120 patients with pain syndromes [40]. Of the 120 patients, 30 were selected as controls. The remaining 90 patients were randomized into three different treatment groups, one of which assessed NK cell activity. All patients in the treatment groups received acupuncture stimulation of ST36 (in conjunction with the LI4 acupoint) with needles retained in place for 30 min. Peripheral blood of the 30 untreated patients and 30 treated patients per each group was examined for the presence of *β*-endorphin and number of NK cells. While untreated patients were examined once, treated patients were examined before, and 30 min and 24 h after acupuncture treatment. The scientists found an increase in *β*-endorphin after 30 min of treatment in the peripheral blood of patients who received acupuncture. More importantly, 30 min and 24 h after acupuncture treatment, they found that the peripheral blood of 40% and 50% of patients, who had low numbers of peripheral blood NK cells before treatment, had increased numbers of NK cells, respectively.

Together, these reviewed studies provide intriguing evidence that acupuncture stimulation modulates NK cell number and function, with the animal studies indicating the immuno-enhancing acupoint (ST36) to be pivotal. For the sake of clarity, we emphasize that we are not claiming this reviewed evidence is conclusive; rather our claim is that there is a sufficiently strong evidential base to warrant theoretical exploration, from a cellular immunology and molecular biological perspective, of possible mechanisms by which acupuncture modulates NK cell number and function. The next section lays the groundwork for such a task by providing an overview of the current understanding of NK cells.

## 4. NK Cells Are Important Effectors of Anticancer Immune Mechanisms

**Figure 1:**
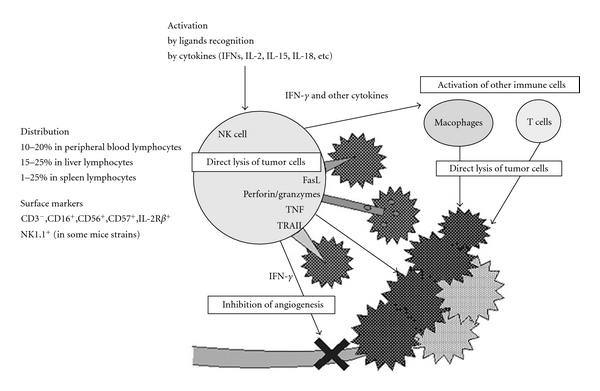
NK cells in tumor surveillance. Reproduced with permission from Takeda and Okumura; *Evid Based Complement Alternat Med*. 2004 : 1 : 17–27.

NK cells are essential effector cells of the innate immune system that spontaneously kill transformed and infected cells, and therefore represent the first line of the host immune defense against cancer and pathogens [41–43]. NK cells utilize two major constitutive mechanisms to recognize and kill cancer cells: the secretory/necrotic mechanism, which is mediated by cytolytic activity of the secreted cytotoxic molecules perforin and granzymes and non-secretory/apoptotic mechanism, which is mediated by transmembrane TNF superfamily ligands (Figure 1) [44, 45]. The secretory/necrotic mechanism is operative against rare leukemia target cells (e.g., in humans, K562 erythroleukemia; in mice, Yac-1 T-cell leukemia) that both express ligands for killer cell activating receptors (KARs) and lack MHC class I molecules. This killing mechanism is induced in NK cells by balanced triggering of KARs (i.e., NKp30, NKp44, NKp46 and NKG2D) with KAR ligands (e.g., the MHC class I homologues MICA and MICB) and disengaging killer cell inhibitory receptors (KIRs) in the absence of MHC class I molecules [46–48]. The non-secretory/apoptotic killing mechanism is operative against all types of cancer cells and is more efficient than the secretory/necrotic mechanism. It is triggered by a simultaneous engagement of the NK cell transmembrane TNF superfamily ligands TNF, FasL and LT-*α*1*β*2 with the corresponding cancer cell transmembrane TNF family receptors TNFR1-TNFR2, Fas and LT-*β*R [42, 44, 45, 49]. These two NK cell cytotoxic mechanisms are operative in both rodents and humans. Because normal cells, as opposed to cancer cells, express relatively high levels of MHC class I molecules and low levels of KAR ligands [50, 51], and do not express the major proapoptotic TNF family receptors TNFR1, TRAILR1 and TRAILR2 [52], these two cytotoxic mechanisms mediate selective killing of tumor cells without harming normal cells. Using these constitutive and cancer-specific cytotoxic mechanisms, NK cells spontaneously and rapidly eliminate newly formed cancer cells and blood born metastases *in vivo*, and prevent development of both primary tumors and metastases [41, 42]. To sum up, NK cells play an all-star role in protecting hosts from carcinogenesis and metastases. 

In addition to KIRs and KARs, NK cells express a variety of other biologically important receptors, including the receptors for Fc portions of immunoglobulin G (Fc*γ*RIII and Fc*γ*RII) and inflammatory and immunoregulatory cytokines [41, 53]. Therefore, NK cells respond not only to transformed and infected cells but also to immune complexes, inflammation and immune reactions by the increases of their constitutive tumoricidal activities and development of proliferation and production of immuno-regulatory cytokines (e.g., IFN-*γ*, TNF and GM-CSF) and chemochines (e.g., IL-8, MIP-1*α*, MIP-1*β* and RANTES) [41, 42, 54–58]. The newly acquired functions may lead to the remarkable augmentation of NK cell anticancer activities and highly increased direct elimination of cancer cells [42], and induction and regulation of cancer-specific Th1 adaptive immune responses [59], which in concert can destroy and control growth of established tumors and metastases. Among the cytokines, IL-2 and IFN-*α* are particularly potent stimulators of NK cells. These two cytokines produce remarkably high growth and tumoricidal activity in NK cells. In response to stimulation by IL-2 and IFN-*α*, NK cells become capable of destroying all types of cancer cells in a highly efficient way, thereby leading to elimination of established tumors and metastases both in rodents and humans [60, 61]. Because of their stimulating ability, IL-2 and IFN-*α* have been approved by the FDA and used with a significant therapeutic efficacy to treat patients with advanced malignant melanoma and/or renal cell carcinoma.

NK cells arise from multipotent hematopoietic stem cells (HSCs) that differentiate through a series of defined phenotypic stages. Precursor and immature NK cells were identified in the bone marrow [62]. Cells with an immature NK cell phenotype were also found in murine liver prompting the suggestion that some peripheral tissues could act as reservoirs for less differentiated NK cell progenitors [63]. In humans, a precursor specified to the NK cell lineage was identified in the lymph node [64]. Like other hematopoietic and immune cells, human NK cells can be fully reconstituted in myeloablated individuals by autologous or allogeneic bone marrow HSCs [65–67]. Mature NK cells can be generated *in vitro* from adult blood and bone marrow cells containing HSCs. Both HSCs and mature NK cells have been found in significant numbers in multiple rodent and human tissues/organs including bone marrow, thymus, blood, spleen, lymph nodes, omentum, intestines, skin, lung and liver. This indicates that NK cells are present and can develop in multiple sites [65–67]. The interaction between membrane-bound ligand LT*α*1*β*2 expressed on HSCs and LT*β*R, expressed on stromal cells, as well as stem cell factor (SCF, c-kit ligand), Flt3-L, IL-7 and IL-15, secreted by stromal cells, provide critical signals that induce differentiation cascades of NK cell development from HSCs [65–67]. The number and function of host NK cells is maintained at required levels by a well defined homeostatic mechanism that is mediated by soluble IL-7 and IL-15R*α*-transpresented IL-15 [68].

There are indications that the CNS may regulate anticancer activities of NK cells via neuropeptides. In this regard, endorphins are the most prominent among neuropeptides. Endorphins are endogenous opioid neuropeptides that are produced by the pituitary gland and hypothalamus during strenuous exercise, high psychological excitement, pain, orgasm and death [69, 70]. *β*-endorphin is a cleavage product of pro-opiomelanocortin (POMC), which is also the precursor of adrenocoticotrophic hormone (ACTH). It is produced and released into the blood stream by pituitary gland and into spinal cord and brain tissues by hypothalamic neurons. NK cells sense and respond to endogenous and exogenous opioids [71–74]. *β*-endorphin enhances both the number and activity of NK cells in the spleens of mice [71]. The effect appears to be mediated via classical opioid receptors, since augmentation of NK cell activity can be inhibited by the antagonist naloxone [71]. *β*-endorphin has also the ability to induce, in mice, increases in the expression of cell adhesion molecules on NK cells and the number of their conjugates with tumor cell targets, as well as the expression of essential cytotoxic molecule of NK cell secretory/necrotic cytotoxicity pathway (granzyme B) [72]. These *β*-endorphin-induced molecular changes might be one of the important mechanisms enhancing NK cell tumoricidal activity [72]. Recent studies show that transplantation into the paraventricular nucleus of hypothalamus of *in vitro* differentiated embryonic hypothalamic neurons producing increased quantities of *β*-endorphin results in significant increases in NK cell tumoricidal activity and NK-cell related resistance to *N*-methyl-*N*-nitrosourea induction of prostate cancers in rats [74]. In humans, *β*-endorphin has also been shown to induceincrease in NK cell cytotoxic activity [73].

In contrast to the above described stimulatory signals that activate and/or expand NK cells and increase their anticancer activity, there is evidence indicating that the CNS and cancer products have the ability to suppress NK cell anticancer functions. For example, strong chronic emotional or physical stress may induce highly increased and/or prolonged secretion of corticosteroids and endorphins which suppress NK cell anticancer functions [75]. Further, high-dose morphine consumption, as well as some cancer products, such as TGF-*β*, FasL, oncoproteins [71, 76, 77] and soluble MIC ligands [78], provide strong inhibitory signals for NK cells that counteract NK cell anticancer functions.

In brief, there is compelling evidence that: cytokines, cancer products and CNS neuropeptides regulate NK cell function, that NK cells regulate both innate and adaptive anticancer immune functions, and that NK cells directly kill cancer cells.

## 5. Molecular Mechanisms of Action

**Figure 2:**
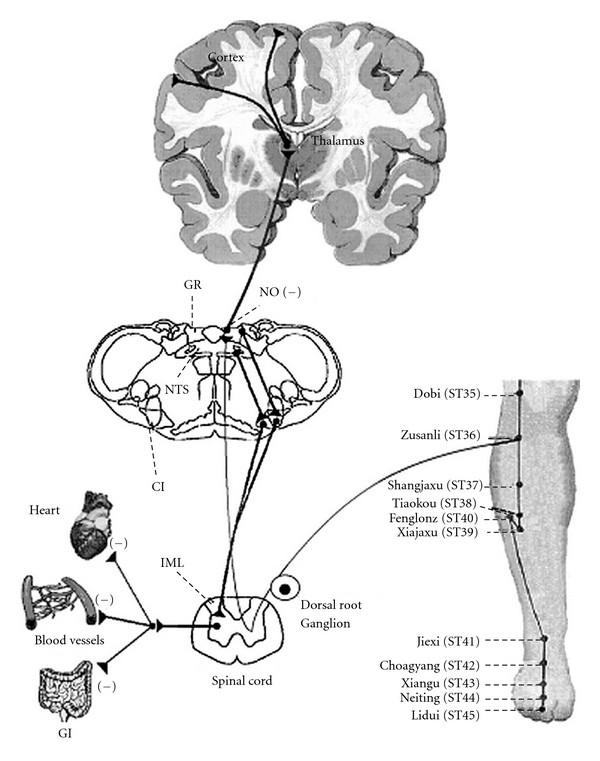
Neural circuits related to somatosympathetic reflexes in the gracile-thalamic-cortex pathways. Reproduced with permission from Ma, *Evid Based Complement Alternat Med* 2004 : 1 : 41–47.

There is a coherent body of research indicating acupuncture initiates a cascade of reactions that stimulates the production and blood-borne dissemination of *β*-endorphins; the research is largely but not exclusively focused on ST36.  Figure 2 traces out the mechanisms by which acupuncture stimulation of ST36 activates the neurotransmitter network in the brain. At the cellular level, needling ST36 induces the enzyme nitric oxide synthase in keratinocytes [79, 80]. In response, these skin cells produce the neurotransmitter nitric oxide [79] which sends signals via the spinal cord to the brain [34, 81, 82]. Simultaneously, NO can also directly stimulate NK cells and induce increases in NK cell tumoricidal activity and proliferation [83]. In the brain, acupuncture stimulation of ST36 elicits widespread and synchronized signals in the cerebro-cerebellar circuit; this is especially marked in the limbic system, which plays a central role in the regulation of immunological functions [33]. Acupuncture-induced signals stimulate the hypothalamus-pituitary-adrenal (HPA) axis to release an endogenous opioid neurotransmitter (*β*-endorphin), which travels from the brain via the blood stream to body locations containing immune cells [84]. 

We begin our synthesis with the statement that acupuncture triggers cross-talk between the neurotransmitter network and the immune system that leads to NK cell activation. This cross-talk is anchored by opiod receptors. When the blood-borne *β*-endorphin arrives to lymphoid tissues, it probably binds to opioid receptors expressed on the surface of NK cells [85, 86]. The bound *β*-endorphin stimulates NK cells to increase the expression of cell adhesion molecules, cytotoxic molecules such as granzyme B and perhaps perforin, TNF superfamily ligands [45, 49, 52, 71–73], and the secretion of cytokines such as IFN-*γ* into the cellular microenvironment [87]. These processes directly and indirectly promote NK cell tumoricidal activities and their ability to eliminate tumor cells and control tumor growth. The molecular processes we are describing provide a theoretical framework for the finding of Yamaguchi and colleagues [39] (discussed above) that acupuncture stimulation is associated with an increase in NK cell expression of cytolytic molecules, tumoricidal activity, quantity and IFN-*γ* secretion in humans.

**Figure 3:**
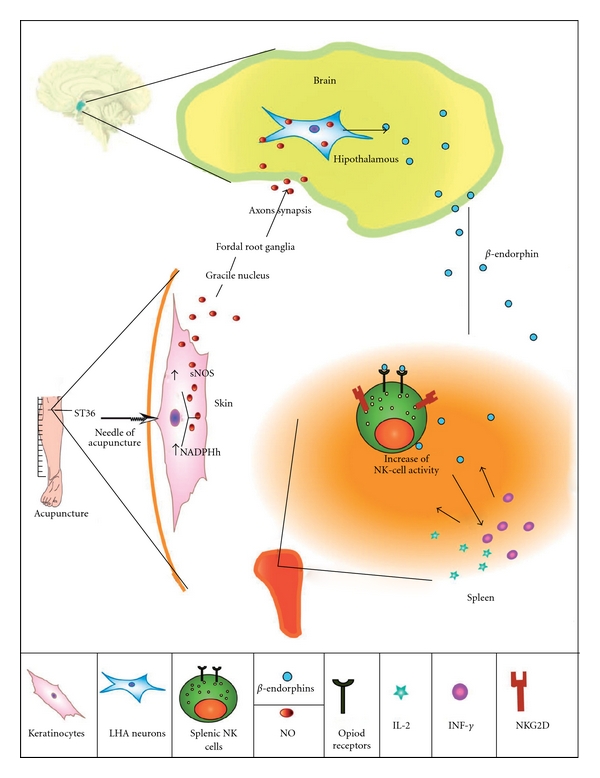
Hypothetic model of the mechanisms how acupuncture stimulates the immune system. Acupuncture stimulation of ST36 acupoint induces release of nitric oxide (NO). NO, a neurotransmiter, stimulates via the sensory nerves, spinal cord and medulla oblongata Gracile nuclceus the lateral hypothalamic area (LHA), where it promotes secretion of opiod peptides such as *β*-endorphin. *β*-endorphin travels via blood circulation to the spleen and other body locations containing immune cells where it binds to opiod receptors expressed on the surface of NK cells and stimulates NK cells to amplify their expression of cytotoxic molecules and consequently tumoricidal activity, and production of IFN-*γ*. This cytokine induces the expression of NK cell receptors and cytokine receptors on NK cells and perhaps cytokine secretion by other immune cells, thereby orchestrating and further amplifying anticancer immune functions.

There is insufficient experimental evidence to precisely identify the cytotoxic mechanisms through which acupuncture stimulation operates to eliminate tumor cells, but we have some clues. It has been reported that *β*-endorphin stimulates NK cells to increase the perforin, granzyme B and IFN-*γ* levels in ethanol treated Fischer rats [88]. Since acupuncture stimulation also activates secretion of *β*-endorphin, we suspect that at least one of the pathways through which NK cells stimulated by acupuncture treatment act to eliminate malignant cells is the perforin/granzyme B-mediated secretory/necrotic mechanism of killing. In addition, IFN-*γ*, induced by acupuncture stimulation, may promote expression of TNF superfamily ligands on NK cells and dendritic cells and their non-secretory/apoptotic mechanism of killing cancer cells mediated by these molecules [44, 49, 52, 83]. Although the scientific community will continue to sort out the specific details of operative pathways over the coming years, we consider there to be sufficient evidence to formally hypothesize that acupuncture stimulation increases the cytotoxic activity of NK cells (Figure 3). 

Besides acting as an incubator for the activation of NK cells, the lymphoid tissues also act as an incubator that facilitates increases in NK cell number, which typically varies from 2 to 18% of the total lymphocytes in human peripheral blood [89]. As described in the previous section, the number of mature NK cells is regulated by continual homeostatic processes that include NK cell precursor proliferation, differentiation and mature NK cell demise. Purine rich box-1 (PU.1) is an important regulatory gene and transcription factor that masters the entire process of NK cell differentiation; it also plays a special role in controlling proliferation during the transition from committed NK cell precursors to immature NK cells [66]. PU.1 is responsible for the regulation of c-Jun. Importantly, acupuncture has been found to increase the expression of c-Jun [90]. Although no directly supporting evidence is available, the finding that acupuncture induces increases in expression of c-Jun, which is regulated by PU-1, suggests that acupuncture might work through the PU.1 pathway to regulate stem cell differentiation and proliferation leading to the increased production of mature NK cells. Proliferation of NK cells may also be linked to IL-2, a cytokine produced by activated T cells and dendritic cells. In the previous paragraph, we pointed out that *β*-endorphin stimulates NK cells to secrete IFN-*γ* into the cellular microenvironment. *β*-endorphin could also simultaneously stimulate activated T cells and dendritic cells to secrete other cytokines such as IL-2 into the cellular microenvironment [87], which would then promote increases in NK cell proliferation and quantity [90].

We continue our synthesis with the statement that acupuncture-induced cross-talk is also anchored by NK-cell receptors. Korean scientists have utilized mRNA microarray tracking to follow gene expression patterns in NK cells that change in response to needling ST36 in a rat model, relative to a sham treatment group and non-treatment group [36]. The study showed acupuncture stimulation of ST36 produced an increase of mRNA CD94 expression, which coded a lectin-like receptor that paired with NKG2 receptors. In turn, CD94 proteins pair with NKG2C to form activating receptors to induce NK-cell activation via ITAM, a tyrosine-based activation motif in NKG2C. The authors point out that CD94 can also simultaneously form heterodimes with NKG2A to block NK cell activity via ITIM, a tyrosine-based inhibitory motif in NKG2A. In this model, however, acupuncture stimulation of ST36 decreased the expression of SHP-1, which encoded a tyrosine phosphatase required for the inhibitory effect of NKG2A. Acupuncture stimulation of ST36 also induced expression of mRNA PTK that encoded the PTK (including Src, Syk and Zap-70 family). PTKs activate downstream proteins that play important roles in multiple cellular processes including proliferation and NK-cell activation. A more recently published, higher quality study confirms many of these findings [91].

Another major NK cell receptor responsible for triggering NK cell cytolytic activity is NKp44 [92, 93], an area investigated in several different ways by different research teams in China. Importantly, the cell surface expression of NKp44 is progressively increased by NK cells during culture with IL-2 [93]. Experimental results demonstrate that acupuncture of ST36 increases IL-2, both in mice [94] and in humans [95, 96] This finding suggests that acupuncture stimulation of ST36 also produces a sequential molecular cascade along the following lines: needling increases secretion of IL-2, which induces an increased expression of NKp44, thereby leading to enhanced NK cell cytolytic activity.

## 6. Assessing the Evidence

To systematically assess the methodological quality of reviewed studies, we turn to a modified version of the Jadad scale [97, 98]. The Jaded scale is the scale most commonly used to assess the quality of research in health studies [99]. White and Ernst modified the Jadad scale specifically to assess acupuncture research [100–102]; their modified Jaded scale ranges from 0 to 5 points with a higher score signifying better methodological quality.

Results from the three human studies are statistically significant and consistent but the methodological quality is low, ranging from 1 to 2 points (Table 2). One of the primary problems is that the studies lacked either a control group or randomization to a control group. Consequently it is possible that changes in NK cell activity and level may result partially or even completely from reasons other than a direct physiological or psychological connection with acupuncture; such alternative reasons include spontaneous remission, the natural course of waxing and waning of symptoms, and regression to the mean [103]. In contrast, the methodological quality of the cited animal studies is higher ranging from a Jaded score of 2–5. Looking specifically at the evidence concerning the idea that acupuncture stimulation activates NK cells (Table 3), we observe that the evidence is consistently supportive across all three studies. All three studies assign animals to a treatment group, placebo control group and non-placebo control group; yet the assignment is not random and therefore some of the critiques presented above hold (although not as strongly as with humans because animals are bred to be similar). Evidence regarding the idea that acupuncture stimulation leads to proliferation of NK cells has a similar profile (Table 4). Evidence concerning the idea that acupuncture stimulation modulates expression of NK cell receptors is produced from research designs that are much stronger (Table 5).

## 7. Future Studies

In drawing upon cellular immunology and molecular biological theory to develop an evidence-based hypothesis that acupuncture enhances anticancer immunity, we provide a new framework for considering anew important, unresolved issues, some of which are specific to CAM and others that are more interdisciplinary in nature. Within the field of CAM, there is considerable interest in identifying the extent to which stimulating specific acupoints produces meaningful results beyond the generic effects that accrue from inserting an acupuncture needle into any point on the body. Authors of a Cochrane review, which synthesizes 40 trials involving 4858 patients, conclude that stimulating the P6 acupoint, compared to sham, significantly reduces nausea, vomiting, and the need for rescue antiemetics [25]. Yet another review, one that examines acupuncture studies across a variety of conditions, concludes that clinical trials have failed to demonstrate a difference in effects between sham and true acupuncture [104]. This review provides a new venue to consider this issue of point specific effects, a venue with an outcome that can be objectively and quantitatively assessed. An advocate of generic effects would seemingly expect that administering acupuncture to any single site on the body would increase NK cell cytotoxicity and number. Or at best that needling any one of a number of different acupuncture points would improve NK cell function and quantity in equivalent ways. For example, one of the anonymous reviewers suggested that a point with a segmental relation to the lungs could be very important because the lungs contain significant amounts of NK cells and lymphoid tissues. We read the literature to imply (without conclusively demonstrating) that needling ST36 (compared to both sham and true points) would increase the number and cytotoxicity of NK cells in humans. In many of the animal studies, needling ST36 alone does produce such an effect [35, 36, 92]. That said, we also consider it likely that needling ST36 in conjunction with other well-chosen points would lead to even more effective immune enhancement. Indeed, the human studies we reviewed utilized point combinations [38–40]. Factors that may modulate the extent to which acupuncture improves the quantity and function of NK cells include the nature of the clinician-patient encounter [105], the intensity of acupuncture stimulation [106], and whether or not the clinician achieves “de qi” (achieving a sensation of stimulation after needle insertion [107]).

Another CAM-specific item is that acupuncture likely has beneficial impact upon various other immunological functions including T-cells [40, 108, 109] and B-cells [32]. This review is focused on NK cells, but we consider it likely that scientists could draw upon biomedical concepts to theorize the ways in which acupuncture stimulation enhances T cells, B cells and other immune functions. Such reviews would not be trivial and would likely entail entirely new research papers.

At a much broader level, this review opens up new lines of inquiry about acupuncture and cancer. As of yet, there are no experimental studies directly investigating whether acupuncture stimulation prevents cancer or produces antitumor effects. Such studies could proceed along the following lines. Mice would be randomly assigned to treatment, placebo control and non-stimulation groups. Then, normal or transgenic inbred mice that spontaneously develop cancer would be treated with a carcinogen for study of cancer prevention. Normal inbred mice would be transplanted with syngeneic tumor cells to investigate if the therapy has antitumor effects. The treatment groups would receive needle stimulation, the placebo control at another point and the non-stimulation group would not receive any needle stimulation. The mice would be followed for carcinogen-induced tumor appearance or tumor transplant growth and tumor-induced death, respectively. In addition, the mice would be examined for NK cell number and function. At a set period of time, the group results would be compared, thereby yielding either supportive or disconfirming evidence.

An inter-disciplinary item of great importance is the extent to which acupuncture could complement conventional therapies to improve patient outcomes. The human studies that we reviewed are based upon research with, respectively, anxious women, healthy volunteers and people suffering pain. It is important to specifically examine the extent to which acupuncture stimulation comparably enhances immune functioning in patients with the multifactorial disease known as cancer, especially since this population of patients is known to have decreased NK activity [110]. Positive results would, in our opinion, provide strong rationale for concerted efforts to integrate acupuncture into conventional cancer care with the aim of increasing survival and disease-free survival of patients with cancer.

## 8. Conclusion

In this review, we have synthesized heterogeneous research findings with cellular immunological and molecular biological theory to produce the “acupuncture immuno-enhancement hypothesis”. Broadly, the hypothesis proposes that acupuncture enhances the ability of the immune system to more actively eliminate malignant cells by increasing the ability of NK cells to kill cancer cells. More precisely, the hypothesis trumpets that acupuncture stimulation increases the cytotoxicity of NK cells by promoting cross talk between the neurotransmitter network and immune system that is [1] orchestrated by nitric oxide, *β*-endorphin and cytokines and [2] anchored by opioid and NK cell receptors. We intend the “acupuncture immuno-enhancement hypothesis” to provide a focal point for future communication and research about the potential of acupuncture to enhance anticancer immune functions.

## Funding

National Institutes of Health (Grant No. RO1 DE17150) to N.L.V.

## Figures and Tables

**Table 1:** Changes of CD16^+^ and CD56^+^ NK cells in peripheral blood following acupuncture.

Timing of measurement	CD16^+^ (%)	CD56^+^ (%)
Before acupuncture	0.8 ± 0.2	5.8 ± 0.7
First day after acupuncture	1.6 ± 0.3*	7.5 ± 1.0*
Second day after acupuncture	2.2 ± 0.5*	8.5 ± 1.5*
Eighth day after acupuncture	2.1 ± 0.4*	11.2 ± 1.8**

Data are means  ±  SD. Blood samples were drawn from the forearm vein with a syringe containing heparin in 17 healthy volunteers aged 21–51 years who received one acupuncture treatment on August 10, 2005. The mononuclear blood cells were separated, stained with fluorescent conjugated monoclonal antibodies and analyzed with fluorescence-activated cell sorter. Table adopted from Yamaguchi et al. [39].

*P < .05, **P < .01: statistical significance of differences of the results compared with baseline levels.

**Table 2:** Studies with evidence that acupuncture stimulation modulates NK cell function in humans.

Authors, journal, year of publication	Arranz et al., *Am J Chin Med*, 2007	Yamaguchi et al., *eCAM*, 2007	Petti et al., *J Trad Chin Med*, 1998
Subjects	Women with anxiety (controls, healthy women)	Normal individuals	Patients with pain
Treatment group	Yes	Yes	Yes
Placebo control group	No	No	No
Non-placebo control group	Yes	No	Yes
Overall Jaded score^a^	2	1	1
Scored component items of the Jaded score			
Study described as randomized (Yes = 1; No = 0)	0	0	1
Randomization appropriate (Yes = 1; No = −1)	0	0	−1
Subject blinded (Yes = 1; No = 0)	0	0	0
Evaluator blinded (Yes = 1; No = 0)	1	0	1
Subjects that withdrew and/or dropped out described (Yes = 1; No = 0)	1	1	0

^a^Used to assess quality of methods, with 5 being the highest score possible.

**Table 3:** Studies with evidence that acupuncture stimulation activates NK cells.

Authors, journal, year of publication	Yu et al., *J Neuroimmunol*, 1998	Yu et al., *Jpn J Physiol*, 1997	Choi et al., *Neurosci Letter*, 2002
Subjects	BALBrc_qrq.mice, athymic BALBrc and ICR	Inbred F344 rats	Sprague-Dawley rats
	*nu*r*nu* mice, LE rats and New Zealand white rabbits		
Treatment group	Yes	Yes	Yes
Placebo control group	Yes	Yes	Yes
Non-placebo control group	Yes	Yes	Yes
Overall Jaded score^a^	2	3	2
Scored component items of the Jaded score			
Study described as randomized (Yes = 1; No = 0)	0	0	0
Randomization appropriate (Yes = 1; No = −1)	0	0	0
Subject blinded (Yes = 1; No = 0)	1	1	1
Evaluator blinded (Yes = 1; No = 0)	1	1	1
Subjects that withdrew and/or dropped out Described (Yes = 1; No = 0)	0	1	0

^a^Used to assess quality of methods, with 5 being the highest score possible.

**Table 4:** Studies with evidence that acupuncture stimulation leads to proliferation of NK cells.

Authors, journal, year of publication	Kim et al., *J Neuroimmunology*, 2005	Yu et al., *J Neuroimmunology*, 1998	Choi et al., *Neurosci Lett*, 2002
Subjects	Sprague-Dawley rats	BALBrc_qrq.mice, athymic BALBrc and ICR	Sprague-Dawley rats
		*nu*r*nu* mice, LE rats and New Zealand white rabbits	
Treatment group	Yes	Yes	Yes
Placebo control group	No	Yes	Yes
Non-placebo control group	Yes	Yes	Yes
Overall Jaded score^a^	3	2	2
Jaded score components			
Study described as randomized (Yes = 1; No = 0)	0	0	0
Randomization appropriate (Yes = 1; No = −1)	0	0	0
Subject blinded (Yes = 1; No = 0)	1	1	1
Evaluator blinded (Yes = 1; No = 0)	1	1	1
Subjects that withdrew and/or dropped out described (Yes = 1; No = 0)	1	0	0

^a^Used to assess quality of methods, with 5 being the highest score possible.

**Table 5:** Studies with evidence that acupuncture stimulation modulates expression of NK cell receptors.

Authors, journal, year of publication	Rho et al., *Mol Cells*, 2008	Kim et al., *J Neuroimmunology*, 2005	Xiao et al., *Chen Tzu Yen Chiu*, 1992	Ma et al., *Zhen Ci and Yan Jiu*, 1992	Wu et al., *Chung-Kuo Chung Hsi i Chieh Ho Tsa Chih*, 1994
Subjects	Sprague-Dawley rats	Sprague-Dawley rats	People with rheumatoid arthritis (controls, healthy people)	BALB/c mice	Hospital patients diagnosed with a malignant tumor prior to surgery
Treatment group	Yes	Yes	Yes	Yes	Yes
Placebo control group	No	No	Yes	No	Yes
Non-placebo control group	Yes^b^	Yes	Yes	Yes	Yes
Jaded score^a^	5	3	2	3	3
Jaded score components					
Study described as randomized (yes = 1; no = 0)	1	0	1	1	1
Randomization appropriate (yes = 1; no = −1)	1	0	−1	1	−1
Subject blinded (yes = 1; no = 0)	1	1	0	1	1
Evaluator blinded (yes = 1; no = 0)	1	1	1	1	1
Subjects that withdrew and/or dropped out described (yes = 1; no = 0)	1	1	1	1	1

^a^Used to assess quality of methods, with 5 being the highest score possible.

^b^Authors state “At the same time, the control group of rats (*n* = 6) were restrained in holders without EA stimulation or with EA stimulation at a non-acupoint”.
